# Acrometastasis in renal cell carcinoma: a case report and literature review^؆^

**DOI:** 10.1016/j.abd.2025.501232

**Published:** 2025-11-01

**Authors:** Emre Zekey, Gülcan Saylam Kurtipek, Murat Çelik

**Affiliations:** a Department of Dermatology, Meram State Hospital, Konya, Turkey; b Department of Dermatology, Faculty of Medicine, Selcuk University, Konya, Turkey; c Department of Pathology, Faculty of Medicine, Selcuk University, Konya, Turkey

*Dear Editor,*

Acrometastasis is an exceptionally rare manifestation of bone metastasis, involving the distal regions of the extremities, such as the hands and feet, and accounting for only about 0.1% of all skeletal metastases.^1^ Despite the relatively high incidence of bone metastases in advanced stages of solid malignancies, the occurrence of metastases in acral locations remains uncommon. Its diverse and often nonspecific presentation may mimic infections, inflammatory disorders, or traumatic injuries, leading to misdiagnosis or delayed recognition. In some cases, patients may initially present to dermatology or orthopedic clinics with localized symptoms, prompting further investigation and revealing an underlying malignancy. The rarity and diagnostic challenges associated with acrometastasis underscore the importance of maintaining clinical suspicion in patients with unusual acral symptoms, particularly when there is a known history of cancer. Early identification, although unlikely to significantly alter the overall poor prognosis, can inform appropriate palliative or therapeutic strategies.

A 51-year-old male patient presented to our dermatology outpatient clinic with a 3–4 month history of swelling, erythema, and pain localized to the distal phalanx of the right thumb. The patient had a known history of renal cell carcinoma (RCC) and was under active oncological surveillance.

Dermatological examination revealed a firm, purplish-red swelling encompassing the distal phalanx, accompanied by erosion of the nail folds, thickening of the nail plate, discoloration, and irregular nail morphology (Fig. 1A). Radiographs revealed marked osteolytic destruction and loss of structural integrity in the distal phalanx (Fig. 1B).

**Fig. 1:**
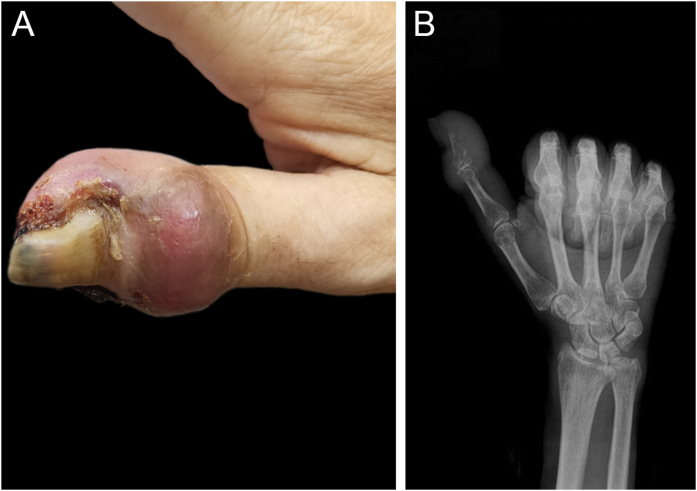
(A) In the distal phalanx of the right thumb, there was a purplish-red swelling encircling the finger, accompanied by erosions of the nail folds, thickening of the nail plate, discoloration, and morphological irregularities. (B) Radiographic imaging revealed extensive bone destruction and a loss of the anatomical integrity of the distal phalanx.

Histopathological evaluation of the lesion revealed neoplastic infiltration characterized by nests and sheets of tumor cells embedded in a desmoplastic stroma. The tumor cells had oval nuclei, a clear eosinophilic cytoplasm, and prominent nucleoli. Focal areas displayed rhabdoid morphological features (Fig. 2A–C). Immunohistochemical staining revealed strong, diffuse positivity for pan-Cytokeratin (panCK), vimentin, and CD10 and negative staining for Cytokeratin-7 (CK7) and Cytokeratin-20 (CK20). Collectively, these findings confirmed the diagnosis of acrometastasis secondary to RCC. Unfortunately, the patient died of a myocardial infarction before the biopsy results were completed, highlighting the aggressive nature of the disease.

**Fig. 2:**
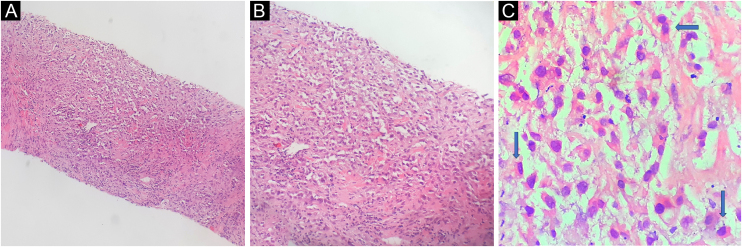
(A) Tumor nests and layers within desmoplastic stroma (Hematoxylin & eosin, ×100). (B) Tumor cells with oval-round nuclei and clear cytoplasm (Hematoxylin & eosin, ×200). (C) Rhabdoid morphology seen in focal areas (blue arrows) (Hematoxylin & eosin, ×400).

Renal cell carcinoma (RCC) is the most prevalent form of kidney cancer in adults. Skeletal metastases are observed in up to 30% of patients with advanced RCC, significantly compromising quality of life due to debilitating bone pain. Acrometastases, a rare variant of bone metastasis, are frequently associated with a poor prognosis. Acrometastases localized to the hands account for approximately 0.1% of all metastatic cases. Primary malignancies commonly implicated in the development of acrometastases include lung, kidney, breast, and colorectal cancers.^2^, ^3^

Typical presentations of acrometastasis include erythema, swelling, pain, limited range of motion, and involvement of the nails and adjacent structures. Lesions are most commonly localized to the distal phalanx of the dominant hand, particularly in the third digit.^4^ However, in the case presented here, the lesion was confined to the distal phalanx of the right thumb.

The pathogenesis of acrometastasis remains incompletely understood. Given that the majority of acrometastases are associated with lung cancer, it has been hypothesized that neoplastic cells disseminate primarily through hematogenous routes rather than via the lymphatic system.^5^ Several factors, including increased blood flow, repetitive trauma, bone marrow density, and local cytokine release, have been proposed as potential facilitators of tumor cell colonization at acral sites.^6^, ^7^, ^8^ Furthermore, enhanced heparanase activity in clear cell renal cell carcinoma (RCC) may promote metastasis by stimulating osteoclastic activity.^9^, ^10^ The upregulation of Receptor Activator of Nuclear factor Kappa-B (RANK) and its Ligand (RANKL) in renal cell carcinomas has been implicated in both enhanced osteoclast-mediated bone resorption and the facilitation of metastatic progression. Notably, RANKL protein has been demonstrated to promote migratory capacity in clear cell renal cell carcinoma cell lines under in vitro conditions.^11^, ^12^

The differential diagnosis of acrometastasis should include chronic paronychia, arthritis, osteomyelitis, trauma, gout, and osseous Paget's disease. Limited follow-up and complex prediagnostic procedures may lead to delayed or incorrect diagnoses, adversely affecting treatment outcomes. Given the poor prognosis of acrometastasis, timely imaging and histopathological evaluation are essential. Histopathology remains key for definitive diagnosis; in our case, the biopsy revealed clear cell carcinoma with rhabdoid morphology, indicating a more aggressive course.^13^

Although acrometastases are uncommon, they should be considered in the differential diagnosis of painful and treatment-resistant digital lesions, especially in individuals with a known history of cancer. Histopathological and immunohistochemical analyses remain the gold standard for establishing a definitive diagnosis. Early identification can assist in guiding therapeutic strategies; however, the overall prognosis generally remains poor.

## ORCID IDs

Emre Zekey: 0000-0001-6237-1534; Gülcan Saylam Kurtipek: 0000-0002-3106-4280; Murat Çelik: 0000-0002-0798-1310

## Authors' contributions

Emre Zekey: The study concept and design; data collection, or analysis and interpretation of data; writing of the manuscript or critical review of important intellectual content; data collection, analysis and interpretation; effective participation in the research guidance; intellectual participation in the propaedeutic and/or therapeutic conduct of the studied case; critical review of the literature; final approval of the final version of the manuscript.

Gülcan Saylam Kurtipek: Writing of the manuscript or critical review of important intellectual content; analysis and interpretation; effective participation in the research guidance; intellectual participation in the propaedeutic and/or therapeutic conduct of the studied case; critical review of the literature; final approval of the final version of the manuscript.

Murat Çelik: Data collection, or analysis and interpretation of data; intellectual participation in the propaedeutic and/or therapeutic conduct of the studied case; critical review of the literature; final approval of the final version of the manuscript.

## Statement of ethics

Ethical approval is not required for this study in accordance with local or national guidelines. Written informed consent was obtained from the patient for publication of the details of their medical case and any accompanying images.

## Financial support

None declared.

## Research data availability

Does not apply.

## Conflicts of interest

None declared.
